# A Review on The Pathogenesis of Cardiovascular Disease of *Flaviviridea* Viruses Infection

**DOI:** 10.3390/v16030365

**Published:** 2024-02-27

**Authors:** Tie-Hua Yang, Wen-Cong Gao, Xin Ma, Qian Liu, Pan-Pan Pang, Yong-Tang Zheng, Yinnong Jia, Chang-Bo Zheng

**Affiliations:** 1 School of Chinese Materia Medica, Yunnan University of Chinese Medicine, Kunming 650500, China; y15974744680@163.com (T.-H.Y.); ap15912401668@163.com (P.-P.P.); 2 Key Laboratory of Animal Models and Human Diseases Mechanisms of Chinese Academy of Sciences, Center for Biosafety Mega-Science, Kunming Institute of Zoology, Chinese Academy of Sciences, Kunming 650201, China; m_xin15@163.com (X.M.); zhengyt@mail.kiz.ac.cn (Y.-T.Z.); 3 Engineering Laboratory of Peptides of Chinese Academy of Sciences, Key Laboratory of Bioactive Peptides of Yunnan Province, KIZ-CUHK Joint Laboratory of Bioresources and Molecular Research in Common Diseases, Center for Biosafety Mega-Science, Kunming Institute of Zoology, Chinese Academy of Sciences, Kunming 650223, China; 4 School of Pharmaceutical Science and Yunnan Key Laboratory of Pharmacology for Natural Products, Kunming Medical University, Kunming 650500, China; gao_wc@126.com; 5 College of Modern Biomedical Industry, Kunming Medical University, Kunming 650500, China; 6 School of Pharmacy, Chongqing Medical University, Chongqing 400016, China; liuqian_670515@163.com; 7 Yunnan Vaccine Laboratory, Kunming 650500, China

**Keywords:** *Flaviviridae* viruses, *Flavivirus*, *Hepacivirus*, cardiovascular disease

## Abstract

Members of the *Flaviviridae* family, encompassing the *Flavivirus* and *Hepacivirus* genera, are implicated in a spectrum of severe human pathologies. These diseases span a diverse spectrum, including hepatitis, vascular shock syndrome, encephalitis, acute flaccid paralysis, and adverse fetal outcomes, such as congenital heart defects and increased mortality rates. Notably, infections by *Flaviviridae* viruses have been associated with substantial cardiovascular compromise, yet the exploration into the attendant cardiovascular sequelae and underlying mechanisms remains relatively underexplored. This review aims to explore the epidemiology of Flaviviridae virus infections and synthesize their cardiovascular morbidities. Leveraging current research trajectories and our investigative contributions, we aspire to construct a cogent theoretical framework elucidating the pathogenesis of *Flaviviridae*-induced cardiovascular injury and illuminate prospective therapeutic avenues.

## 1. Introduction

Cardiovascular disease reigns as the preeminent cause of global mortality, exhibiting a persistently escalating annual death toll. Among the multifarious etiologies, viral pathogens, such as coxsackieviruses, influenza viruses, human herpes viruses, human immunodeficiency viruses, and the emergent acute respiratory syndrome coronavirus, have been recognized as significant contributors to cardiovascular morbidity [1,2,3]. *Flaviviridae* viruses significantly impact human health [4] and have spread and become endemic in more than 100 countries and regions, with hundreds of millions of people being infected each year and many experiencing severe symptoms, making *Flavivirus* infections a serious public health problem worldwide [5]. This review is poised to dissect the cardiovascular implications of *Hepacivirus* and *Flavivirus* infections within the *Flaviviridae* cohort. Our objective is to elucidate their potential nexus with cardiovascular disease, thereby furnishing pivotal insights that could catalyze the identification of novel prophylactic and therapeutic targets against cardiovascular ailments associated with *Flaviviridae* viruses.

## 2. *Flaviviridae* Viruses

**Figure 1:**
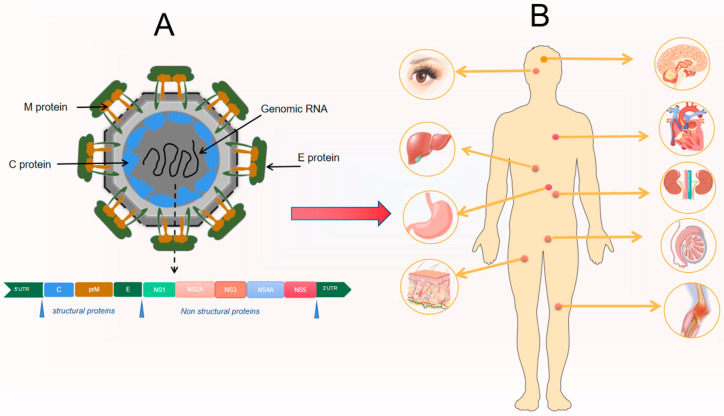
Flaviviridae Viruses Structure and Associated Systemic Damage. (**A**) Flaviviridae, a single-stranded RNA virus, comprises three main structural proteins: envelope (E), membrane (M), and capsid (C), along with several nonstructural proteins. (**B**) Infection with Flaviviridae viruses can lead to diverse systemic damage, including encephalitis in the nervous system, hepatitis in the digestive system, reproductive complications, and urinary system disorders.3. *Flaviviridae* viruses and cardiovascular disease.

The *Flaviviridae* family of viruses are enveloped viruses with a single-stranded RNA genome that is ortho-polar in nature. This family consists of four genera: *Flavivirus, Hepacivirus, Pestivirus,* and *Pegivirus* [6]. *Flavivirus* is capable of being transmitted by mosquitoes and ticks and can infect humans, causing a range of clinical symptoms, including mild fever, severe hemorrhagic fever, and even nerve invasion. There are over 70 viruses within the *Flavivirus* genus, including well-known viruses such as Zika virus (ZIKV), West Nile virus (WNV), dengue virus (DENV), and yellow fever virus (YFV). *Hepacivirus*, another genus within the *Flaviviridae* family, includes the blood-borne human hepatitis C virus (HCV). The Pestivirus genus includes the bovine viral diarrhea virus (BVDV) and classical swine fever virus (CSFV) [7]. *Hepatovirus* and *Flavivirus,* both genera within the *Flaviviridae* family, have been reported to have a significant impact on human health [7]. Over the past few decades, there has been a continuous increase in the global incidence of *Flavivirus* infections, with large outbreaks occurring in various regions of the world [8]. This expansion has gone beyond endemic tropical and subtropical regions and is attributed to factors such as a lack of vector control, inadequate investment in healthcare, increased regional travel and migration flows, global warming, deforestation, and rapid urbanization. Certain *Flaviviridae* viruses have been found in infected animals, as well as multiple organ tissues in humans, and have been clinically associated with cardiovascular disease (Figure 1).

## 3. *Flaviviridae* Viruses and Cardiovascular Disease

### 3.1. HCV

Identified in 1989, HCV stands as the exclusive member of the *Hepacivirus* genus within the *Flaviviridae* family [9]. It casts a long shadow on global health, with disease burden estimates indicating an alarming 5.8 million fatalities attributable to HCV over a decade [10]. The ramifications of HCV are profound and far-reaching, with it being a primary etiological factor in liver fibrosis, hepatocellular carcinoma, and mortality, thus significantly exacerbating the global morbidity and mortality landscape [11].

Accumulating evidence underscores a paradigm shift in the perception of HCV infection, from a traditionally liver-centric ailment to a systemic disease with potent extrahepatic manifestations, including cardiovascular disease. Studies clearly indicate an elevated cardiovascular risk in HCV-infected individuals, with a cardiovascular mortality rate reported at 1.39% in this cohort [12]. HCV is implicated in fostering atherosclerosis and thrombosis, as evidenced by Vassalle et al.’s revelation of a heightened prevalence of coronary artery disease among HCV patients [13]. Moreover, a subsequent study documented a 1.43-fold escalated risk of peripheral arterial disease in HCV-infected subjects compared to their non-infected counterparts [14]. Beyond these vascular complications, HCV is also linked with an array of cardiac pathologies, including myocarditis, dilated and hypertrophic cardiomyopathies, as well as arrhythmogenic right ventricular cardiomyopathy [15,16]. Notably, one investigation highlighted a potential association between HCV and right ventricular systolic dysfunction, along with the onset of pulmonary hypertension [17]. This was further substantiated by the significantly higher levels of myocardial injury biomarkers, such as N-terminal brain natriuretic peptide precursor (NT-proBNP) and circulating cardiac troponin, observed in HCV patients [18]. Complementing these findings, Australian research has reported a marked diminution in left ventricular end-diastolic volume and per-breath output in HCV-infected individuals, affirming the virus’s detrimental impact on cardiac function [19].

**Figure 2:**
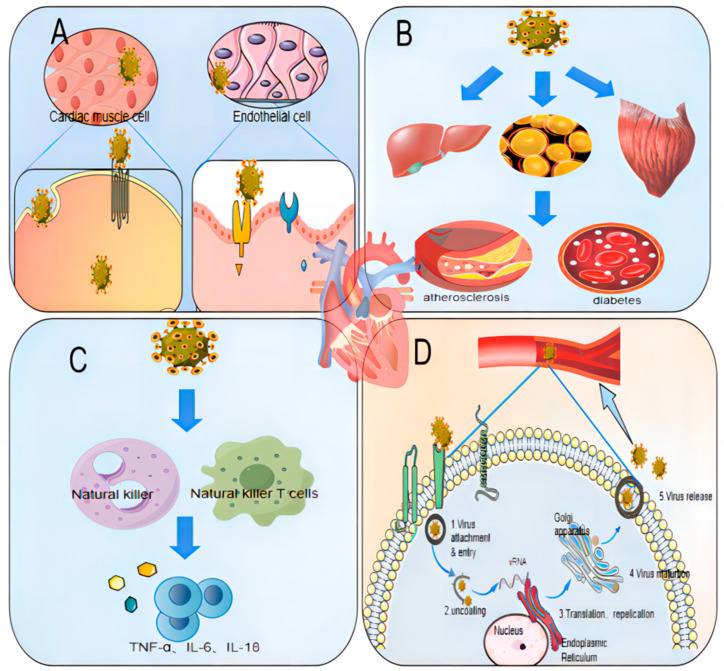
Pathways of HCV-Induced Cardiovascular Disease. (**A**) Direct Viral Damage: HCV has the capacity to invade and replicate within cardiac and vascular tissues, leading to direct cellular injury. (**B**) Metabolic Imbalance: HCV infection can impair liver function, disrupting cellular lipid and energy metabolism. This disruption contributes to the development of atherosclerosis and diabetes mellitus, both of which are risk factors for cardiovascular disease. (**C**) Immune-Mediated Damage: Infection with HCV stimulates the systemic immune response, exacerbating cardiovascular damage through immune-mediated mechanisms. (**D**) Viremia: Persistent replication of HCV within the host increases the viral load, which in turn contributes to cardiovascular impairment.

Extant research delineates several mechanisms by which HCV infection may precipitate cardiovascular pathology (Figure 2). Primarily, HCV directly impinges upon the cardiovascular system, colonizing and proliferating within carotid plaques and cardiomyocytes. This invasion engenders vascular inflammation, atherosclerosis, and cardiac fibrosis [20]. Furthermore, HCV core proteins exacerbate cardiovascular disease by elevating reactive oxygen species (ROS) in cardiomyocytes, prompting the release of cytochrome c from the inner mitochondrial membrane and activating cysteine asparaginase, culminating in genomic DNA fragmentation and subsequent cardiomyocyte apoptosis [21]. The infection also provokes a robust inflammatory response, exacerbating cardiovascular disease via systemic inflammation and oxidative stress. HCV incites an innate immune reaction mediated by natural killer (NK) cells and natural killer T (NKT) cells, releasing critical effectors like tumor necrosis factor (TNF-a) and interleukin 6 (IL-6). This process disrupts the TNF-α/IL-10 and IL-6/IL-10 ratios, fostering localized inflammation and fibrosis [22]. Elevated TNF-a further inhibits l-type Ca^2+^ channel currents, diminishing peak myocardial systolic Ca^2+^ concentrations and adversely affecting cardiomyocyte contraction [23]. Moreover, high levels of inflammatory factors contribute to vascular dysfunction. HCV RNA interacts with the toll-like receptor (TLR)-3 on endothelial cells, provoking IL-6 release and endothelial inflammation, a precursor to atherosclerosis [24]. Additionally, inflammatories like IL-8 and chemokine10 (CXCL10) promote endothelial cell apoptosis, further impairing vascular function [25,26]. HCV’s impacts extend to metabolic processes, disrupting lipid and glucose metabolism and insulin signaling pathways, thereby increasing the prevalence of hyperlipidemia, hepatic steatosis, insulin resistance, metabolic syndrome, and diabetes mellitus—all recognized as significant cardiovascular disease risk factors [27]. Acute and chronic HCV infections alter plasma lipid levels, meddling in lipid and glucose metabolism within host cells. HCV promotes lipid production and accumulation by damaging the liver and inhibiting microsomal triglyceride transfer protein (MTP) activity essential for very-low-density lipoprotein (VLDL) assembly in hepatocytes [28], alongside impeding mitochondrial ATP production [29,30]. Moreover, HCV proteins upregulate TNF-α expression, hinder insulin receptor substrate (IRS-1/2) and Akt phosphorylation, and stimulate gluconeogenic gene expression, fostering insulin resistance and culminating in hyperglycemia and hyperlipidemia [30]. Additionally, studies have shown that hemodialysis patients with high and moderate HCV viremia exhibit a significantly increased left ventricular mass (LVM) index compared to those with low viremia and controls [31]. This heightened LVM index correlates with elevated cardiovascular risk factors, such as ventricular end-diastolic diameter, end-systolic diameter, and ventricular septal thickness, suggesting viremia’s contributory role in predisposing individuals to cardiovascular disease.

### 3.2. YFV

Yellow fever (YF) is a severe acute hemorrhagic fever induced by the YFV. The virus is believed to have its origins in Africa and is presently endemic to 34 African countries and 13 South American nations. Annually, YF accounts for approximately 200,000 cases in these regions, leading to approximately 20,000 fatalities [32]. Despite the availability of an efficacious vaccine, the nature of its mosquito-borne transmission renders complete eradication unfeasible. Consequently, yellow fever continues to pose a significant public health challenge in both Africa and South America [33,34].

YF predominantly targets the liver, but it can also adversely affect other tissues, including the cardiovascular system. Historically recognized in 1882 for its capacity to impact cardiovascular health, YF is notably characterized by bradycardia, a condition where the heart rate is slower than normal [35]. Further investigations have revealed more about YFV’s deleterious effects on the cardiovascular system. An increase in eosinophils surrounding the heart in YFV infections suggests impaired myocardial function [36]. Moreover, cases of imported yellow fever have documented myocardial damage. Electrocardiogram (ECG) abnormalities, particularly sinus bradycardia, have been observed in over half of the patients with yellow fever [35]. Post-mortem examinations of heart tissue from individuals who succumbed to YFV have disclosed extensive cardiac damage, including myocardial necrosis, inflammatory infiltration, acute endocarditis, epicarditis, and thrombosis in coronary artery branches [37]. These findings collectively underscore the significant impact of YFV on the cardiovascular system and highlight the necessity of further research to understand and mitigate these effects.

**Figure 3:**
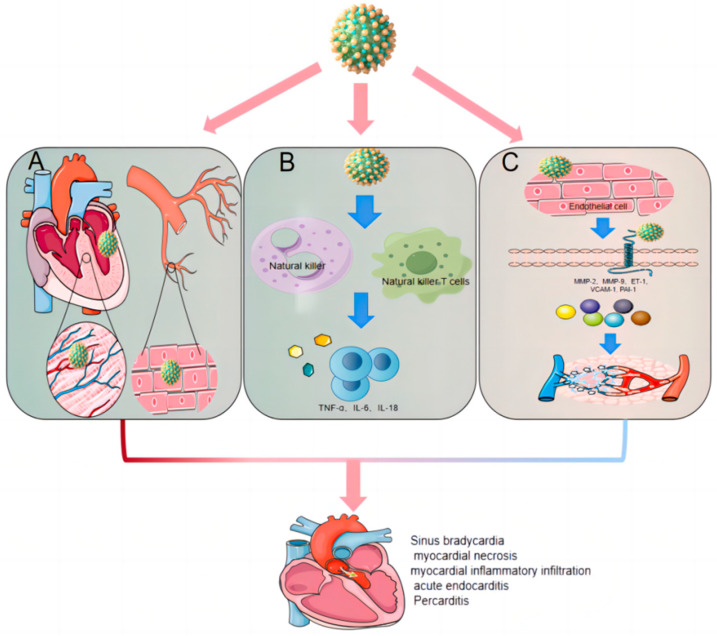
Mechanisms Underlying YFV-Induced Cardiovascular Disease. (**A**) Direct Viral Damage: YFV antigens are detectable within cardiac and vascular tissues, suggesting a direct pathogenic effect on these systems. (**B**) Immune-Mediated Damage: YFV infection activates systemic immunity, leading to an “inflammatory storm”. This heightened immune response subsequently results in cardiovascular system damage. (**C**) Vascular endothelium Impact: Infection with YFV elevates levels of matrix metalloproteinases (MMP-2, MMP-9) and endothelial markers (ET-1, VCAM-1), culminating in endothelial injury and increased vascular permeability.

Contemporary research indicates that the cardiovascular damage wrought by YFV infection is multifactorial, stemming from direct viral effects, inflammatory processes, and vascular endothelial lesions (Figure 3). Upon entering the host through a mosquito bite, YFV is conveyed by dendritic cells to lymphoid tissues for initial replication, subsequently disseminating via the bloodstream to internal organs and tissues, like the myocardium and endothelium, precipitating cardiac and vascular dysfunction [38]. The detection of YFV antigens in the hearts of infected individuals and the accompanying myocardial damage indicate a direct viral assault on cardiomyocytes [39]. Inflammation is pivotal in YFV-associated cardiovascular injury. Typically, infected cells secrete the type I interferon (IFN), which activates the Jak/STAT pathway and induces the expression of antiviral genes [40,41,42]. However, YFV’s non-structural protein 5 (NS5) undermines this response by inhibiting a signal transducer and transcription factor 2 (STAT2), impairing the host’s IFN response and facilitating prolonged viral persistence [42]. Additionally, YFV prompts monocytes and macrophages to release a deluge of pro-inflammatory mediators, like IL-6, IL-2, and TNF-α, inciting a ‘cytokine storm’ where the severity of heart failure correlates with the level of inflammatory factors [43]. CXCL10, upregulated by YFV infection, further aggravates cardiac hypertrophy and insufficiency [44]. Hemorrhagic fever, a hallmark of YFV-induced cardiovascular disease, arises from capillary damage and plasma leakage. Endothelial function deterioration is pivotal here, with YFV infection escalating the expression of endothelial damage biomarkers, such as angiopoietin-2 (Ang-2), matrix metallopeptidase 2 (MMP-2) and MMP-9, endothelin-1 (ET-1), acetyl tetrapeptide-1 (syndecan-1), vascular cell adhesion molecules1 (VCAM-1), and plasminogen activator inhibitor-1 (PAI-1) [45,46,47]. Elevated endothelin-1 levels, in particular, heighten inflammation and cytokine (IL-1β, TNF-α, and IL-6) production, disrupting the endothelial barrier and inducing hemorrhage. Higher levels of syndecan-1, endothelin-1, and VCAM-1 are also associated with a worse prognosis in sepsis [45], underscoring their contributory role in exacerbating cardiovascular disease. This intricate interplay of viral action, inflammation, and endothelial damage underscores the complexity of YFV’s impact on the cardiovascular system.

### 3.3. DENV

DENV stands as a prominent arbovirus, with estimates suggesting that DENV-induced outbreaks potentially impact up to 4 billion individuals worldwide, representing nearly 50% of the global population [48]. At present, DENV infections have been documented in over 128 countries, placing a significant socioeconomic and public health burden internationally [49]. Consequently, the management and containment of the dengue epidemic demand ongoing and comprehensive vigilance from the global health community.

Approximately 12.5% of patients with severe dengue fever reportedly suffer from cardiovascular complications, manifested as abnormal heart rates, hypotension, myocarditis, pericarditis, and impaired myocardial function [50,51]. A study conducted in Taiwan revealed that 68.6% of individuals infected with DENV experienced acute adverse cardiovascular events, primarily ischemic stroke and heart failure [52]. Further emphasizing the cardiovascular impact, Dinesh Kumar Yadav and colleagues discovered that 70% of children with severe dengue exhibited abnormal cardiac function index (Tei), indicative of both systolic and diastolic dysfunction. This condition potentially predisposes them to myocarditis and heart failure [53]. Beyond its direct cardiac effects, dengue fever is also implicated in vascular dysfunction [54]. Research indicates that DENV infection can trigger endothelial inflammation and compromise the vascular endothelial glycocalyx layer. Such alterations lead to vascular dysfunction and potentially expedite the progression of vascular diseases [55,56]. These findings underscore the profound and multifaceted impact of DENV on cardiovascular health, highlighting the need for further research and improved clinical strategies to mitigate these serious complications.

**Figure 4:**
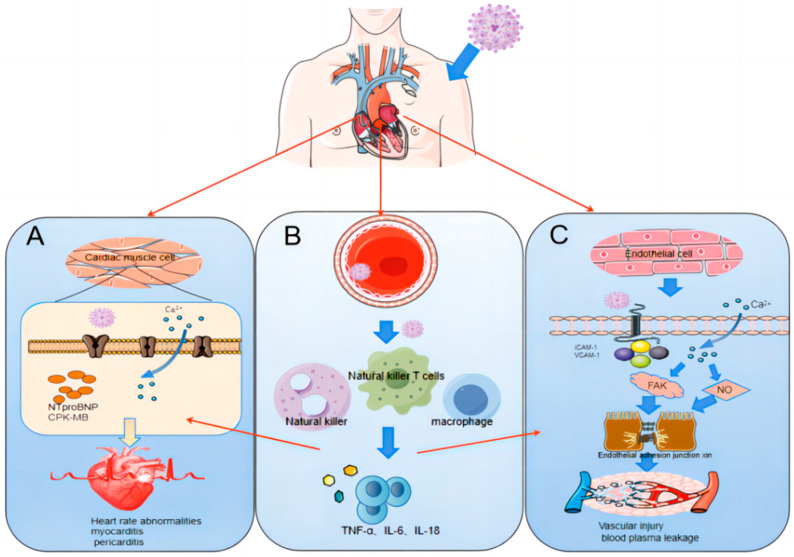
Mechanisms of DENV-Induced Cardiovascular Disease. (**A**) Direct Cardiac Damage: DENV infection is associated with elevated levels of NT-proBNP, CK-MB, and Ca^2+^ in cardiac tissues. This elevation is indicative of cardiomyocyte damage and can predispose patients to arrhythmias. (**B**) Immune-Mediated Damage: The infection triggers a systemic immune response, characterized by the release of cytokines and interleukins (IL-1, IL-2, IL-6, etc.), tumor necrosis factor, and histamine. This cascade leads to inflammation and necrosis of vascular endothelial cells, thereby contributing to cardiovascular impairment. (**C**) Endothelial Dysfunction: DENV infection results in increased cytoplasmic Ca^2+^ levels in endothelial cells, promoting the production of nitric oxide and prostacyclin. Concurrently, it activates FAK expression in endothelial cells. This disrupts the adherens junction proteins within the endothelium, undermining the structural integrity of the endothelial barrier.

The pathogenesis of cardiovascular lesions in dengue fever is a critical area of investigation, with contemporary studies highlighting viral targeting [57], immune response, and endothelial dysfunction as key factors associated with post-infection cardiovascular manifestations (Figure 4). Direct cardiovascular system infection by DENV is evident, as the virus has been detected in human and animal cardiomyocytes and vascular endothelial cells [58,59]. Elevated levels of cardiomyocyte damage markers, such as troponin, NT-proBNP, and creatine kinase MB (CK-MB), as well as increased levels of soluble intercellular adhesion molecules1 (sICAM-1) and VCAM-1 in endothelial cells and circulating endothelial cells (CECs), indicate direct cardiovascular injury by DENV [60,61]. Calcium ions (Ca^2+^), pivotal for cardiac and circulatory function, are also implicated in DENV pathogenesis. DENV infection is associated with reduced serum levels of free Ca^2+^ in patients and an increase in Ca^2+^ ion inflow in myocytes at rest, which may contribute to DENV-induced arrhythmias and altered myocardial contractility [59,62]. Inflammatory mediators play a crucial role in cardiovascular injury. Upon DENV entry into the host, T cells and macrophages are activated, releasing an array of inflammatory substances, including cytokines and interleukins (IL-1, IL-2, IL-6, etc.), tumor necrosis factor, and histamine [63,64]. The resultant release of inflammatory factors like IL-1β and TNF-a results in vascular endothelial cell lining inflammation and necrosis, increasing capillary permeability and vascular leakage [65]. Vascular leakage in myocyte interstitial spaces triggers myocardial interstitial edema, impairing myocardial contraction and overall function [23]. Endothelial dysfunction is another facet of DENV’s impact on cardiovascular health. The interaction between the DENV NS1 protein and the glycocalyx layer of the vascular endothelium increases capillary permeability [66]. DENV infection elevates cytoplasmic Ca^2+^ levels in endothelial cells, leading to nitric oxide and prostacyclin production and activating the expression of adhesion kinase (FAK) in the endothelium. This disrupts endothelial adhesion junctional proteins and compromises endothelial barrier integrity. Furthermore, DENV affects mitochondrial function in endothelial cells, enhancing ROS production, promoting viral replication, and inducing endothelial cell death, thereby increasing endothelial permeability and inducing plasma leakage [67]. This multifactorial pathogenesis underscores the complex interplay between DENV and cardiovascular damage, highlighting the necessity for ongoing research and targeted therapeutic strategies.

### 3.4. ZIKV

ZIKV was first identified in the Zika forest of Uganda in 1947 and subsequently detected in human blood in 1952 [68]. By 2019, indigenous transmission of ZIKV has been reported in nearly 90 countries globally [69]. This situation underscores the necessity for heightened surveillance, robust healthcare infrastructure, and effective public health strategies to mitigate the potential health implications of ZIKV spread.

Emerging evidence suggests that ZIKV is also associated with more serious cardiovascular complications. A prospective study has established a link between acute-phase ZIKV infection and cardiovascular complications, suggesting a potential role of ZIKV in the development of cardiovascular disease [70]. Case studies have documented patients with no prior cardiac history who developed arrhythmias, heart failure, pericardial thickening, pericardial effusion, and elevated cardiac enzymes following ZIKV infection [71]. Such findings highlight the virus’s capability to precipitate severe cardiac issues even in those previously unaffected. Additionally, post-ZIKV infection increases D-dimer levels, which may predispose individuals to venous thromboembolic diseases [72]. These revelations underscore the potential for ZIKV to contribute to a spectrum of cardiovascular conditions and highlight a deeper understanding of its pathogenesis and implications for affected populations.

**Figure 5:**
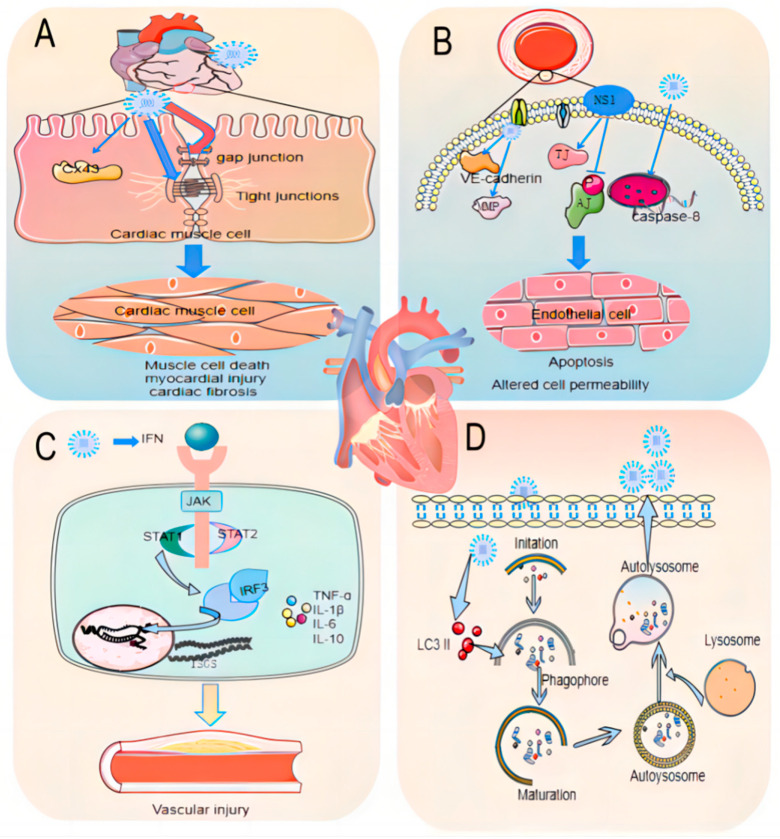
Mechanisms of ZIKV-Induced Cardiovascular Diseases. (**A**) Direct Cardiac Impact: ZIKV infection is linked to increased cardiac myosin levels and down-regulation of Cx43 protein in cardiac tissues. This disrupts the gap junctions and intercalated disc structures between cardiomyocytes, leading to cardiomyocyte damage and potential cardiac dysfunction. (**B**) Endothelial Dysfunction: In endothelial cells, ZIKV infection results in reduced VE-calmodulin expression and heightened MMP activity. These changes disrupt endothelial homeostasis, culminating in vascular dysfunction. (**C**) Immune Response Activation: ZIKV infection triggers the body’s type I and type II interferon (IFN) pathways, leading to the release of ISGs and an increase in pro-inflammatory factors, thereby elevating the risk of cardiovascular complications. (**D**) Autophagy-Related Cardiac Injury: ZIKV facilitates myocardial injury by promoting viral replication via up-regulation of autophagy-related LC3B expression in cardiac cells.

ZIKV-induced cardiovascular lesions are hypothesized to stem from viral tropism and the host’s inflammatory response to cardiac and vascular tissues (Figure 5). Upon transmission via a mosquito bite, ZIKV replicates in dendritic cells and disseminates through the bloodstream, directly infecting cardiomyocytes and leading to cell death, myocardial injury, fibrosis, and dysfunction [71]. The severely impaired myocardial function in ZIKV-infected hearts is characterized by increased cardiac enzyme levels, down-regulation of the Cx43 protein, and disruption in gap junctions and intercalated discs between cardiomyocytes [73]. ZIKV also infects primary endothelial cells from various blood vessels [74], reducing VE-cadherin expression and increasing matrix metalloproteinase (MMP) expression, thereby altering endothelial homeostasis and permeability and inducing vascular dysfunction [75]. Transcriptome analysis suggests that ZIKV activates apoptotic pathways in endothelial cells via increased caspase-8 transcription, promoting cell death and further vascular dysfunction [75]. The ZIKV NS1 protein stimulates the phosphorylation of adherens junction proteins in human brain microvascular endothelial cells and inhibits tight junctions, exacerbating endothelial dysfunction [76]. The ZIKV infection activates the type I/II interferon pathway, increasing the expression of interferon-stimulating genes (ISGs) through the JAK/STAT signaling pathway. This activation in cardiac and vascular tissues post-infection induces cardiomyocyte apoptosis/sclerosis and impairs vascular cell reproduction and migration, contributing to cardiovascular injury [77,78]. Elevated levels of ISG15 can also affect vasoconstriction and dilation [79]. ZIKV infection increases the release of pro-inflammatory factors (TNF-α, IL-1β, IL-6, IL-10, COX-2) and chemokines (CXCL10, CXCL1, CXCL12, CCL2, CCL4), with excessive pro-inflammatory factors raising the risk of atrial fibrillation in patients [80]. Recent evidence indicates that autophagy plays a role in cardiovascular complications following ZIKV infection. ZIKV activates autophagic activity in cells and inhibition of autophagy reduces vertical transmission of ZIKV, limiting placental damage and fetal death [81]. Autophagy levels in mouse cardiomyocytes significantly increase post-infection, and inhibiting autophagy reduces viral load in cardiomyocytes, mitigating ZIKV infection and its adverse complications [82]. Conversely, the ZIKV non-structural protein NS5 interacts with the cellular protein Ajuba to inhibit PINK1-Parkin-dependent mitochondrial autophagy, inducing pro-inflammatory chemokine production. Restoring cellular mitochondrial autophagy reduces tissue damage by decreasing ZIKV-induced inflammation and invasive effects [83]. Therefore, the role of autophagy in ZIKV-induced cardiovascular injury warrants further exploration.

### 3.5. WNV

WNV, a mosquito-borne *flavivirus*, was first identified in 1937 in a febrile woman in Uganda. Primarily transmitted through the bite of infected mosquitoes [84], it has established itself as a significant public health concern due to its expanding geographic range and the number of infections. In 2018, Europe alone recorded nearly two thousand cases of WNV infection, surpassing the total cases documented in the preceding seven years [85]. Currently, WNV is endemic in regions such as Africa, Europe, the Middle East, and Asia [86], and its continued spread poses an escalating threat to global public health.

Myocarditis and related myocardial pathologies in mammals and birds following WNV infection have been well-documented, indicating the potential of the virus to induce cardiac complications in animals [87]. While initial human cases connecting WNV to myocarditis or cardiomyopathy were sparse, the surge in WNV infections has highlighted its ability to cause myocardial damage in humans as well. WNV infection has been associated with serious cardiac conditions, including fatal arrhythmias, myocardial dysfunction, elevated cardiac enzymes, ventricular tachycardia, ventricular fibrillation, and heart block [88]. In support of this, one study revealed evidence of myocardial damage in patients with WNV infection, characterized by elevated myocardial enzyme levels and electrocardiogram ST segment changes, indicating cardiac dysfunction. The study also suggested that WNV-associated myocardial infection could lead to myocardial infarction [89]. Additionally, a case involving a 69-year-old patient, displayed arrhythmias, myocardial dysfunction, and elevated cardiac enzymes, further suggesting a link between WNV infection and myocardial injury [90]. In another analysis, patients with pre-existing cardiovascular disease experienced exacerbated cardiovascular injury post-WNV infection [91]. Ashley R. Gao documented cases where WNV patients exhibited mild troponin elevation, reduced ventricular exercise function, and decreased ejection fraction, symptoms associated with acute compensated heart failure [92]. These findings collectively underscore the emerging recognition of WNV as a potential contributor to a range of cardiovascular issues in infected individuals.

While the precise mechanisms by which WNV infection influences cardiovascular disease remain largely unreported in the literature, chronic infection and the persistent presence of WNV in host tissues are theorized to be primary contributors to complications in advanced cardiovascular conditions. Studies indicate that WNV can reside in the kidney for extended periods, potentially leading to chronic kidney disease, which could indirectly impact cardiovascular health [93]. Nevertheless, the direct mechanisms impacting the cardiovascular system are yet to be fully elucidated. In addition to chronic infection, the host’s inflammatory response post-infection is believed to be crucial in disease pathogenesis. Acute WNV infection triggers the production of various cytokines, chemokines, and other factors essential for antiviral immunity [8]. Specifically, the activation of IRF-3 and NF-κB signaling pathways by TLR7 and TLR3 following WNV infection leads to the release of proinflammatory cytokines, such as TNF-α, IL-6, IL-1β, and IL-12. These cytokines play a significant role in the pathogenesis of both symptomatic and asymptomatic cases of WNV infection [94]. In the context of cardiovascular disease, such systemic inflammation may exacerbate pre-existing conditions or initiate new pathologies. Further research is necessary to understand the specific pathways and interactions through which WNV affects the cardiovascular system, which will be crucial for developing targeted treatments and preventive strategies.

## 4. Prevention and Treatment of *Flaviviridae* Viruses-Associated Cardiovascular Disease

The management and therapeutic approaches for cardiovascular diseases associated with *Flaviviridae* virusse infections are relatively underdeveloped. Nonetheless, drawing from the synthesis provided, direct antiviral therapy has been identified as the principal strategy for mitigating the advancement of cardiovascular diseases linked to these infections. Currently, while specific antiviral medications targeting Flaviviridae virus infections are lacking, several antiviral agents such as Acyclovir, Ribavirin, Sofosbuvir, and Hydroxychloroquine have demonstrated the ability to inhibit viral replication during infection periods [95,96], indicating their potential as feasible treatment options. Beyond the direct antiviral interventions, the management of the host immune response is a crucial aspect of the treatment strategy. The production of cytokines, notably TNF-α and IL-6, is a significant precipitant of cardiovascular pathology. Therefore, the employment of immunosuppressive agents, including corticosteroids (e.g., Prednisolone), Siltuximab, and Tocilizumab, may play a vital role in ameliorating the progression of cardiovascular diseases following viral infection [97,98]. Moreover, meticulous cardiovascular function monitoring, including assessments of heart rate, blood pressure, and platelet count, facilitates the timely detection and management of cardiovascular abnormalities, such as arrhythmias, hypotension, and thrombosis. In instances of severe arrhythmias, the prompt consideration for the implantation of a temporary pacemaker is advised. In the case of low cardiac output, the administration of inotropic agents and vasopressors is recommended to rectify this condition, thereby alleviating cardiovascular symptoms and ensuring patient health and safety [99]. Overall, a comprehensive approach encompassing direct antiviral therapy, modulation of the host immune response, targeted cytokine inhibition, and vigilant cardiovascular monitoring is necessary to effectively prevent and treat *Flaviviridae* virus-associated cardiovascular disease.

## 5. Conclusions and Future Perspectives

*Flaviviridae* virus infections continue to pose a significant global public health challenge, as these viruses are spreading beyond their traditional tropical and subtropical endemic areas to new regions in the northern hemisphere. This expansion presents a major obstacle to global healthcare systems. *Flaviviridae* viruses have a strong affinity for the cardiovascular system, causing damage and leading to unfavorable short- and long-term outcomes for patients (Table 1). Consequently, addressing the impact of these viruses on human health remains a critical concern. Furthermore, cardiovascular abnormalities caused by *Flaviviridae* viruses remain prevalent in both children and adults, and the study of their pathogenesis is conducive to reducing the incidence of viral cardiovascular diseases and improving the quality of life of patients. Therefore, it is important to investigate the mechanisms of *Flaviviridae* infection and the development of cardiovascular diseases, to analyze the occurrence of cardiovascular diseases associated with *Flaviviridae* viruses infections, to explore clinical manifestations, diagnostic approaches, treatment measures, and prognosis, and to establish effective preventive and treatment guidelines based on these studies. These efforts will help mitigate the cardiovascular injuries caused by *Flaviviridae* virus infections and alleviate the burden of cardiovascular diseases associated with these viruses.

## Figures and Tables

**Table 1:** *Flaviviridae* viruses infection and cardiovascular disease manifestations.

Virus Name	Cardiovascular Disease	Cardiovascular Pathogenic Mechanism
HCV	Atherosclerosis, thrombosis, myocarditis, myocardial hypertrophy, cardiac arrhythmia, myocardial fibrosis	Viral direct damage [20], viremia [31], organismal immunityviraemia [22], metabolic imbalance [27]
YFV	Sinus bradycardia, myocardial necrosis, myocardial inflammatory infiltration, acute endocarditis, Percarditis, and coronary branch thrombosis	Myocardial cell necrosis [38], immune response [42], endothelial dysfunction [45]
DENV	Heart rate abnormalities, hypotension, myocarditis, pericarditis, heart failure, cardiac systolic and diastolic dysfunction, endothelial inflammation	Virulence [59], immune response [64], endothelial dysfunction [65]
ZIKV	Myocarditis, pericarditis, heart failure, arrhythmias, perivascular and endothelial injury, venous thrombosis	Virulence [70], inflammatory pathway [75], autophagy [81]
WNV	Arrhythmias, myocardial dysfunction, elevated cardiac enzymes, ventricular tachycardia, and ventricular fibrillation	/

## Data Availability

Not applicable.
